# College Prevention: A View of Present (and Future) Web-Based Approaches

**Published:** 2011

**Authors:** Scott T. Walters, Clayton Neighbors

College campuses in the United States may be the most electronically “wired” environments on earth. College students use the Internet not only to write term papers and receive correspondence but also to report (and keep track of) friends’ personal status, download music, view classroom lectures, and receive emergency messages. In fact, college students spend considerably more time online than the average person. In a recent survey of U.S. college students (Jones et al. 2009), nearly all respondents (94 percent) stated that they spent at least 1 hour on the Internet each day, with the main tasks including social communication, entertainment, and class work. In keeping with this trend, Web-based programs that address alcohol consumption among college students have become widely available in the United States. This sidebar provides an overview of currently available programs as well as of the advantages and disadvantages of this approach and the future outlook of Web-based programs.

## Overview

Web-based programs that address alcohol consumption among college students now are widely used on U.S. college campuses. Alcohol 101-plus, AlcoholEdu, Alcohol-Wise, and e-CHECKUP TO GO are among the most popular Web-based alcohol prevention programs in the United States (see the table for a list of Web sites linking to these and other programs.) Alcohol 101-plus, a free program created by the Century Council, has distributed starter kits to over 2,500 U.S. universities. AlcoholEdu is a commercially available program distributed by the for-profit company Outside the Classroom and is self-described as being used on hundreds of campuses and by 36 percent of all first-year students at America’s 4-year higher-education institutions. e-CHECKUP TO GO is commercially available from the nonprofit San Diego State University (SDSU) Research Foundation. The program’s Web site notes that it is used internationally on over 550 campuses. Other programs, such as web-BASICS, currently are under evaluation and have been limited to distribution at only a few campuses.

At least two companies (i.e., Outside the Classroom and Third Millennium Classrooms) offer different versions of their programs for primary and secondary prevention. For instance, Third Millennium Classrooms markets programs for primary prevention (e.g., all incoming freshmen), secondary prevention (e.g., fraternities and sororities), and disciplinary referrals. Although the most widely available Web-based programs are focused on alcohol, at least two programs deliver marijuana-specific content, including the Marijuana e-CHECKUP TO GO (SDSU Research Foundation) and Marijuana 101 (Third Millennium Classrooms).

Program content, format, and length vary considerably, but most programs include at least some content derived from empirically supported interventions, such as normative drinking feedback or expectancy challenge (Carey et al. 2009; Moreira et al. 2009). Programs also usually provide tools for calculating blood alcohol concentrations (BACs) and information regarding effects at different BAC levels. Finally, programs often include information regarding family history, short-term risks, and tolerance.

Web-based programs are used in different contexts. For example, students may be asked to complete a Web-based program as part of new-student orientation, as a class requirement (e.g., for health and wellness classes), or to fulfill an organizational requirement (e.g., fraternity and sorority alcohol education). Other students may be required to complete a Web-based program as a sanction for violating campus alcohol policy.

## Advantages and Disadvantages

There are clear advantages and disadvantages to Web-based programs compared with traditional face-to-face programs (Cunningham 2009). For example, Web-based programs are easier to implement and disseminate than in-person approaches. They also may be cost-effective to colleges because they do not require staff training, scheduling, or allocation of interview space and time. In addition, they are convenient for students because they do not require students to rearrange their schedules to complete the program. Finally, continuing technological advances have and will continue to expand the possibilities of Web-based approaches. Most programs already use a variety of components such as video, interactive feedback, extensive branching, and content that can be tailored on the basis of individual or group characteristics. For instance, abstainers, moderate drinkers, and heavy drinkers each may receive different messages, which may in turn increase the impact of the program.

The disadvantages of these programs have been less widely recognized. Although it is possible that the relative anonymity of Web-based interventions facilitates more accurate reporting, it is equally conceivable that some students will feel less accountable to provide quality responses. The relative lack of effort needed to complete some Web-based programs, compared with face-to-face interventions, also may affect attributions of the value of the information received—that is, students may assign less value to interventions that take relatively little effort to complete. Web-based programs also may be completed in the context of multiple distractions (e.g., while the respondent is texting, watching television, holding phone conversations, or even drinking) that do not occur in in-person settings. And by making the material “easy” for students to access and complete, the programs can verify the respondent’s engagement to both knowledge questions and (nonmonitored) open-ended question prompts only to a limited extent. Thus, although the programs can provide an engaging experience to students who are interested in the material, it is entirely possible for unmotivated students to complete most programs having learned very little. Therefore, it is important to make the material interesting so that students will want to go beyond the bare minimum.

Finally, it is important to acknowledge that the dissemination of these programs has far exceeded the relatively modest evidence base. A recent review of 35 studies (Carey et al. 2009) found that computer-delivered interventions tended to reduce drinking compared with assessment-only control groups but generally produced equivalent results when they were tested in comparison with alcohol-relevant comparison groups, such as alcohol education classes. The studies reviewed also had several limitations. For instance, most studies only involved follow-up periods of about 5 to 6 weeks after the intervention, and relatively few studies used longer-term followups. Moreover, many of the programs evaluated either were not commercially available or involved substantial additional components, such as assessment batteries, in-person meetings, or monitored program completion that would not be present in most prevention contexts. Finally, with few exceptions, program completers were undergraduate volunteers who received compensation for their participation.

## The Future of Web-Based Programs

As the capabilities of Web-based programs improve over time, they undoubtedly will become more engaging and effective. One area in which programs can be improved is in terms of tailoring information and activities to the user. In fact, evidence already exists to indicate that some kinds of information can have a greater impact if tailored to the respondent’s gender and drinking severity. In addition, there are theoretical reasons to believe that the content of programs can be effectively tailored to students on the basis of their age, group membership, or other personal interests. Because college drinking largely is a social behavior, it may make sense to customize programs to students on the basis of social-group affiliation. Some programs already provide separate tracks for students according to their location, drinking status, and gender. Many programs further allow students to customize at least some of the content they view based on what they think is interesting.

A second area for improvement is in program content. Just as dissemination of some programs has exceeded their evidence base, many programs still rely heavily on educational content about the effects and consequences of alcohol or other drugs (AODs), despite the fact that AOD education has a poor track record of changing behavior. One exception to this rule is normative-feedback components, which now are included in most programs. The evidence for normative perceptions as a mechanism of change is solid; studies consistently show that students who overestimate drinking norms tend to drink the most and that interventions that successfully correct normative perceptions tend to reduce AOD use (Larimer and Cronce 2007). However, other mechanisms of change have been described in the literature—including AOD expectancies, risk perception, and self-efficacy—that, to date, are less frequently used in Web-based programs. Because each of these constructs predicts substance use, one would expect that programs that effectively address them would be more likely to reduce AOD use. For instance, positive expectancies could be addressed through interactive expectancy “challenge” activities. With regard to risk perception, rather than providing general information about risk, programs might calculate the probability of some undesirable outcome (e.g., health, social, or academic problems) on the basis of present behavior and allow participants to select from a menu of strategies to reduce their risk to a more acceptable level. Self-efficacy could be enhanced by encouraging users to develop their own concrete plans for safe drinking through an interactive process in which the user suggests some strategies, while the program suggests other strategies on the basis of the student’s demographics, affiliation, or goals. Finally, it also might be possible to tailor program material on the basis of mediators such as the respondent’s motives or expectancies associated with AOD use. For example, because some types of AOD-use motives predict greater risk for problems, this information could be presented both as tailored feedback (e.g., risk estimates based on motives) and as an interactive problem-solving activity (e.g., how to achieve those motives without AOD use).

A third, relatively untapped use of Web-based programs is their ability to stimulate self-reflection through the way users enter information. Although most programs do an outstanding job of using questions to tailor material and verify knowledge of information, they do a relatively poor job of using the questions themselves to stimulate thinking. Indeed, there is an intriguing literature on assessment reactivity demonstrating that reductions in drinking can occur as a result of simply answering survey questions (Walters et al. 2009). Considering assessment as a dynamic rather than a passive task may create more opportunities for self-reflection around drinking through the way users enter (and not just receive) information. For example, the next generation of automated programs might regard assessment and information as equally important aspects. A program might ask respondents to report their AOD use and resulting problems on the same calendar. This approach would allow respondents to draw their own conclusions about the relationship between drinking and problems (e.g., that drinking and problems co-occur in time) rather than having this simply pointed out to them. Likewise, programs might ask people to manually calculate their own number of drinks per week or peak BAC, or rehearse their chosen strategies for avoiding high-risk situations, rather than provide this information to the respondents.

Finally, given the popularity of social-networking sites such as Facebook and Twitter, increasing opportunities exist not only to tie prevention programs into social-networking sites but also to present material in the same way as social-networking sites. For instance, students might be able to share thoughts on the material or collaborate on problems. Students might elect to receive follow-up messages that support pro–social drinking or download cell phone applications to assist with drink counting, BAC calculation, or other suggestions for moderating drinking. Consistent with this theme, the National Institute on Alcohol Abuse and Alcoholism recently convened a social-networking brainstorming meeting to consider applications of social-networking programs. Using these and other approaches, Web-based prevention programs can be developed further to reach their full potential in curbing excessive alcohol use among college students.

## Figures and Tables

**Table:** Web sites Targeting College Drinking

**Web sites discussed in this article:** http://www.alcohol101plus.org/home.html http://www.echeckuptogo.com/ http://www.outsidetheclassroom.com/ https://3rdmilclassrooms.com/
**Selected free Web programs/resources:** http://www.alcoholscreening.org/ http://www.collegedrinkingprevention.gov/ http://gordie.org/home.aspx http://www.factsontap.org/ http://rethinkingdrinking.com/

## References

[b1-arh-34-2-222] Carey KB, Scott-Sheldon LA, Elliott JC (2009). Computer-delivered interventions to reduce college student drinking: a meta-analysis. Addiction.

[b2-arh-34-2-222] Cunningham JA, Miller P (2009). Internet evidence-based treatments. Evidence Based Addiction Treatment.

[b3-arh-34-2-222] Jones S, Johnson–Yale C, Millermaier S, Seoane Pérez F (2009). Everyday life, online: U.S. college students’ use of the Internet [article online]. First Monday.

[b4-arh-34-2-222] Larimer ME, Cronce JM (2007). Identification, prevention, and treatment revisited: Individual-focused college drinking prevention strategies 1999–2006. Addictive Behaviors.

[b5-arh-34-2-222] Moreira MT, Smith LA, Foxcroft D (2009). Social norms interventions to reduce alcohol misuse in university or college students. Cochrane Database of Systematic Reviews.

[b6-arh-34-2-222] Walters ST, Vader AM, Harris TR, Jouriles EN (2009). Reactivity to alcohol assessment measures: An experimental test. Addiction.

